# Can Symbiotic Bacteria (*Xenorhabdus* and *Photorhabdus*) Be More Efficient than Their Entomopathogenic Nematodes against *Pieris rapae* and *Pentodon algerinus* Larvae?

**DOI:** 10.3390/biology10100999

**Published:** 2021-10-04

**Authors:** Hanaa Elbrense, Amr M. A. Elmasry, Mahmoud F. Seleiman, Mohammad S. AL-Harbi, Ahmed M. Abd El-Raheem

**Affiliations:** 1 Zoology Department, Faculty of Science, Tanta University, Tanta 31527, Egypt; 2 Botany Department, Faculty of Agriculture, Menoufia University, Shibin El-Kom 32514, Egypt; amr.elmasri@agr.menofia.edu.eg; 3 Plant Production Department, College of Food and Agriculture Sciences, King Saud University, P.O. Box 2460, Riyadh 11451, Saudi Arabia; 4 Department of Crop Sciences, Faculty of Agriculture, Menoufia University, Shibin El-Kom 32514, Egypt; 5 Department of Biology, College of Science, Taif University, P.O. Box 11099, Taif 21944, Saudi Arabia; mharbi@tu.edu.sa; 6 Department of Economic Entomology and Agricultural Zoology, Faculty of Agriculture, Menoufia University, Shibin El-Kom 32514, Egypt; ahmed.abdelraheem@agr.menofia.edu.eg

**Keywords:** entomopathogenic nematodes, *Xenorhabdus* sp., *Photorhabdus* sp., *Pieris rapae*, *Pentodon algerinus*, biocontrol, cytotoxicity

## Abstract

**Simple Summary:**

Food security is the people’s main concern, and agricultural crops play a significant role in ensuring it. Agricultural pests, on the other hand, are regarded one of the most serious threats to cause a significant problem for food security. Entomopathogenic nematodes of the genera *Herterorhabditids* and *Sterinernematids* fulfil the fundamental requirements of perfect bio-control agents; however, their efficacy mostly dependent on their symbiotic bacteria. As a result, this study aimed to investigate the ability of the isolated symbiotic bacteria (*Photorhabdus* and *Xenorhabdus*) to control *Pieris rapae* and *Pentodon algerinus* larvae in comparison with their own nematodes, *Heterorhabditis bacteriophora* and *Steinernema riobravis,* respectively. The results showed that both nematode species and their symbiotic bacteria were able to suppress both insect species. However, both bacterial genera were more efficient than the investigated nematode species against *P. rapae*, although nematodes were superior against *P. algerinus*. Gas chromatography–mass spectrophotometry of *Xenorhabdus* sp. and *Photorhabdus* sp. identified the key components with the insecticidal properties. The two bacteria genera were proven to be safe and had no significant effect on normal WI-38 human cells. In conclusion, the symbiotic bacteria can be employed safely and effectively against the tested insects independently on their own entomopathogenic nematodes.

**Abstract:**

*Pieris rapae* and *Pentodon algerinus* are considered a global threat to agricultural crops and food security; hence, their control is a critical issue. *Heterorhabditid* and *Steinernematid* nematodes, along with their symbiotic bacteria, can achieve the optimal biocontrol agent criterion. Therefore, this study aimed to evaluate the efficacy of *Heterorhabditis bacteriophora*, *Steinernema riobravis,* and their symbiotic bacteria (*Xenorhabdus* and *Photorhabdus*) against *P. rapae* and *P. algerinus* larvae. The virulence of entomopathogenic nematodes (EPNs) was determined at different infective juvenile concentrations and exposure times, while the symbiotic bacteria were applied at the concentration of 3 × 10^7^ colony-forming units (CFU)/mL at different exposure times. Gas chromatography–mass spectrophotometry (GC-MS) analysis and the cytotoxic effect of *Photorhabdus* sp. and *Xenorhabdus* sp. were determined. The results indicated that *H*. *bacteriophora*, *S*. *riobravis*, and their symbiotic bacteria significantly (*p* ≤ 0.001) induced mortality in both insect species. However, *H*. *bacteriophora* and its symbiont, *Photorhabdus* sp., were more virulent. Moreover, the data clarified that both symbiotic bacteria outperformed EPNs against *P. rapae* but the opposite was true for *P. algerinus.* GC-MS analysis revealed the main active compounds that have insecticidal activity. However, the results revealed that there was no significant cytotoxic effect. In conclusion, *H*. *bacteriophora*, *S*. *riobravis*, and their symbiotic bacteria can be an optimal option for bio-controlling both insect species. Furthermore, both symbiotic bacteria can be utilized independently on EPNs for the management of both pests, and, hence, they can be safely incorporated into biocontrol programs and tested against other insect pests.

## 1. Introduction

The cabbage worm, *Pieris rapae* L. (Lepidoptera: Pieridae), and the scarab beetle, *Pentodon algerinus* dispar (Coleoptera: Scarabaeidae), are considered to be among the most important pests that threaten agricultural crops and food security globally. *P*. *rapae* is considered the most common pest of the cruciferous crops, including cabbage, cauliflower, broccoli, and brussel sprouts [1]. *P*. *algerinus* is an endemic in Egypt and the Middle East, and their larvae are called white grubs. Furthermore, they are polyphagous and considered basic pests of different crops, turfgrasses, nurseries, and ornamentals worldwide [1]. They also live in the soil and feed on plant roots [2]. Chemical methods have been used to control both insect pests, but they have not achieved the desired results [3]. Therefore, biocontrolling these pests has become an important priority.

Entomopathogenic nematodes (EPNs) of the *Steinernematid* and *Heterorhabditid* genera are considered among the most important biocontrol agents because of their effectiveness and low cost, as well as their high levels of safety to nontargets. EPNs carry symbiotic bacteria, which have a major role in insect death [4,5,6,7]. Infective juveniles (IJs) of *Heterorhabditid* and *Steinernematid* nematodes actively seek insect hosts, penetrating through an insect’s openings to reach the hemocoel, where symbiotic bacteria in the genera *Photorhabdus* sp. and/or *Xenorhabdus* sp., respectively, are released [8]. Liu et al. [9] reported that the symbiotic bacteria associated with *Steinernematid* and *Heterorhabditid* nematodes were successfully isolated and classified taxonomically both by phenotypic-biochemical criteria and the sequencing of 16S rDNA to *Xenorhabdus* sp. and *Photorhabdus* sp., respectively. They were also identified as Gram-negative bacteria of the family Enterobacteriaceae, having rod shapes and peritrichous flagella. These bacteria can colonize insect hemolymph and degrade insect tissues. They also release several virulence factors, including toxin complexes, hydrolytic enzymes, hemolysins, and antimicrobial compounds that kill insect hosts typically within 48 h [10,11,12]. However, this process provides nutrients for nematode development and reproduction within the insect cadaver. Most of the recent studies have focused on evaluating the efficacy of EPNs in controlling agricultural insect pests [13,14,15,16]. Nevertheless, only a few of these studies have shed light on using the isolated symbiotic bacteria alone for pest control [17,18,19].

The main goal of this study was to find a new approach instead of pesticides to mitigate the hazard impact of both *P. rapae* and *P. algerinus,* which attack agricultural crops. This aim was achieved by evaluating the activity of *S. riobravis* and *H. bacteriophora* against *P*. *rapae* and *P*. *algerinus* in comparison to the activity of their symbiotic bacteria (*Xenorhabdus* and *Photorhabdus*), thus determining whether these symbiotic bacteria can fight the insects independently of their nematodes.

## 2. Materials and Methods

### 2.1. Insects Used in the Current Investigation

Third-instar larvae (2 days old) of *Pieris rapae* and *Pentodon algerinus* were used in this study. *P*. *rapae* was reared in the Entomology Lab, Faculty of agriculture Menoufia University according to Webb and Shelton [20], where butterfly adults were kept in oviposition cages (100 × 100 × 100 cm^3^). Then, they were provided with 10% sucrose solution, and fresh cabbage leaves were continuously provided to favor egg laying. For colony maintenance, egg collection was carried out daily. Subsequently, hatched larvae were provided with fresh cabbage leaves, and emerged pupae were transferred to new rearing cages. Additionally, *P*. *algerinus* third-instar larvae were obtained from the Plant Protection Institute, Dokki, Egypt, where they were reared on potato tubers. Both insects were reared at 30 °C and 12D:12L photoperiods.

### 2.2. Entomopathogenic Nematodes (EPNs)

The two EPNs, namely, *Steinernema riobravis* and *Heterorhabditis bacteriophora*, used in this study were obtained from the Plant Protection Institute, Dokki, Egypt. Nematodes were then mass-reared in the Entomology Lab, Faculty of Science, Tanta University according to Kotchofa and Baimey [21]. Following their protocol, *Galleria mellonella* larvae were exposed to nematode juveniles at a concentration of five juveniles per larva. Then, dead *Galleria* larvae were transferred to white traps for harvesting juveniles [22]. The harvested juveniles were used for the subsequent experiments.

### 2.3. Susceptibility of Third-Instar Larvae of P. rapae and P. algerinus to EPNs, S. riobravis, and H. bacteriophora 

Following Yuksel et al. [23], suspensions of 10, 25, 50, 100, 150, and 200 IJs/mL distilled water of each EPN species were prepared. One milliliter of each suspension was applied to a Whatman’s No. 2 filter paper in a plastic container (9 × 5 cm^2^). Then, ten third-instar larvae of *P*. *rapae* were collected from the colony and introduced into the plastic container containing the treated filter paper. Cabbage leaf discs were provided as food. A distilled water treatment was used as control. Each treatment was replicated five times. 

For *P*. *algerinus*, the previous procedures were followed. However, equal potato tubers were provided as food. Subsequently, *P*. *rapae* and *P*. *algerinus* larval mortalities were recorded daily, and the dead larvae were dissected to ensure the infections. Next, the mortality data from this bioassay were used to estimate the response curve (Slope, LC_50_, and LC_90_ values) using Probit analysis according to Finney [24].

### 2.4. Isolation of the Symbiotic Bacteria, Photorhabdus sp. and Xenorhabdus sp.

Entomopathogenic bacteria (EB), namely, *Xenorhabdus* sp. and *Photorhabdus* sp., were isolated from the *G*. *mellonella* larval hemolymph infected with *S*. *riobravis and H*. *bacteriophora*, respectively, in the Microbiology Lab, Faculty of agriculture Menoufia University according to the method of Poinar and Thomas [25] modified by Vitta et al. [18]. All work was practiced in an air laminar flow cabinet that was cleaned with 70% alcohol, and the fan motor was left on for 15 min at high speed. Briefly, *G*. *mellonella* larvae were infected with *S*. *riobravis* or *H*. *bacteriophora* at a concentration of five IJs per larva in a plastic Petri dish (15 × 3 cm^2^) at 28 ± 2 °C and 12D:12L photoperiod. After 48 h, the infected *G*. *mellonella* larvae were withdrawn, washed with 70% ethanol and then with distilled water, and finally dried on a filter paper. Subsequently, treated larvae prolegs were incised by a sterile sharp needle to create an influx of the hemolymph that contains *Xenorhabdus* or *Photorhabdus* bacteria. Then, the hemolymph samples were distributed on nutrient agar media in Petri dishes (9 × 3 cm^2^). After 24 h, bacterial colonies were plated on NBTA (i.e., nutrient agar with 0.004% triphenyl tetrazolium chloride and 0.025% bromothymol blue) [26], and the process was repeated every 24 h until the pure isolated colonies were obtained. For the bioassays, the isolated bacterial colonies were inoculated in Luria–Bertani (LB) broth and left to multiply for 48 h at a temperature ranging from 28–30 °C in a shaking incubator at 220 rpm. Finally, the cell concentration was adjusted to 3 × 10^7^ colony-forming units (CFU) per mL [27].

### 2.5. Morphological Differentiation between the Two Types of Symbiotic Bacteria

The primary bacterial cells of *Xenorhabdus* sp. and *Photorhabdus* sp. were stained with a Gram stain to describe them. Then, using the streaking approach described by Fukruksa et al. [27], bacterial colonies were distinguished based on their shape and color change on NBTA and eosin methylene blue (EMB) media.

### 2.6. Susceptibility of the Third-Instar Larvae of P. rapae and P. algerinus to Symbiotic Bacteria Xenorhabdus sp. and Photorhabdus sp.

This experiment was performed as described by Adithya et al. [28], in which cabbage leaves were cleaned, dried, and cut into equal leaf discs. Then, 10 of these leaf discs were impregnated in 2 mL of each bacterial suspension at concentration of 3 × 10^7^ CFU/mL. The treated cabbage leaf discs were then picked up and placed in a plastic container (9 × 5 cm^2^) with filter paper (Whatman number 2). Following that, 10 *P. rapae* larvae were put into the plastic container, which was then covered with a porous lid. In addition, cabbage leaf discs treated simply with bacterial medium were employed in a parallel control. Each treatment was replicated five times. Similar approaches were used for *P. algerinus*, with the exception that equal potato tuber pieces were used as food. Finally, daily mortalities of *P. rapae* and *P. algerinus* larvae were recorded for 96 h following treatment. 

### 2.7. Efficacy and Time-Course Viability of Symbiotic Bacteria (Xenorabdus sp. and Photorabdus sp.) against the Third-Instar Larvae of P. rapae under Field Conditions

A small trial was undertaken during the winter season of 2019 in a cabbage field at the Agricultural Research Farm, Faculty of Agriculture, Menoufia University, Egypt, to assess the efficacy and time-course viability of *Photorhabdus* sp. and *Xenorhabdus* sp. bacteria against *P. rapae* third-instar larvae. Four randomized experimental plots were designed in the field. There were five cabbage plantations in each plot. The first plot’s cabbage plantations were treated with a bacterial suspension of *Photorhabdus* sp. at a concentration of 3 × 10^7^ CFU/mL. Following that, *Xenorhabdus* sp. was used to treat the plantations in the second plot at a concentration of 3 × 10^7^ CFU/mL. The plantations in the third plot, however, were just treated with bacterial medium (positive control). Finally, plantations in the fourth plot served as the untreated negative control group. For bioassay, five cabbage leaves were obtained independently from each plot after one hour of the treatment, transferred to the lab, and then cut into equal discs (3 × 3 cm^2^). Then, ten leaf discs from each plot were added to the 20 starved third-instar larvae of *P*. *rapae* in a plastic container (15 × 10 cm^2^). This step was replicated five times, and *P. rapae* larval mortality was recorded 48 h post exposure to leaf discs from each plot. The dead larvae were then sterilized in 70% ethyl alcohol, and a hemocoel sample from the dead insects was taken and streaked onto a nutrient agar media to determine whether the mortality was due to the presence of bacteria or not. Finally, to estimate the time-course viability of both bacteria, the same procedures described above were followed on the second (24 h), third (48 h), and fourth days (72 h) post treatment.

### 2.8. Gas Chromatography–Mass Spectrophotometry (GC-MS) of Photorhabdus sp. and Xenorhabdus sp. Bacteria

The chemical compositions of *Photorhabdus* sp. and *Xenorhabdus* sp. bacteria were determined using a Trace GC-ISQ mass spectrometer (Thermo Scientific, Austin, TX, USA) with a direct capillary column TG–5MS (30 m × 0.25 mm × 0.25 m film thickness) and a direct capillary column TG–5MS (30 m × 0.25 mm × 0.25 m film thickness). The temperature in the column oven was initially maintained at 50 °C, then increased at a rate of 5 °C/min to 200 °C, and maintained for 2 min. After that, the temperature was raised to 300 °C and kept for 2 min. The injector and MS transfer line temperatures were also kept at 270 and 260 °C, respectively. At a constant flow rate of 1 mL/min, helium was also used as a carrier gas. The solvent delay was 4 min, and diluted samples of 1 µL were automatically injected using an Autosampler AS1300 and a split mode GC. EI mass spectra were also taken in full scan mode at 70 eV ionization voltages spanning the *m*/*z* 50–650 range. The temperature of the ion source was fixed to 250 °C. Finally, the main components were identified by comparing their retention durations and mass spectra to the mass spectral databases WILEY 09 and NIST 14.

### 2.9. Cytotoxicity of the Symbiotic Bacteria, Xenorhabdus sp. and Photorhabdus sp.

#### 2.9.1. Cell Lines and Chemical Reagents

The cell line human lung fibroblast (WI-38) was obtained from ATCC via a holding company for biological products and vaccines (VACSERA), Cairo, Egypt. Moreover, RPMI-1640 medium, MTT, and dimethyl sulfoxide (DMSO) (Sigma Co., St. Louis, MO, USA), as well as fetal bovine serum (GIBCO, Loughborough, UK) reagents, were used.

#### 2.9.2. MTT Assay

The purpose of this assay was to see if *Xenorhabdus* sp. and *Photorhabdus* sp. bacteria had any effect on the viability of human lung fibroblast (WI-38) cells. This colorimetric assay is based on the conversion of yellow tetrazolium bromide to a purple formazan derivative by mitochondrial succinate dehydrogenase in viable cells. Cell lines were cultured in RPMI-1640 medium with 10% fetal bovine serum. The antibiotics added were 100 units/mL penicillin and 100µg/mL streptomycin at 37 °C in a 5% CO_2_ incubator. The cell lines were seeded in a 96-well plate at a density of 10^4^ cells/well at 37 °C for 48 h under 5% CO_2_. After incubation, the cells were treated with bacteria and/or medium and incubated for 24 h. Subsequently, 20 µL of MTT solution at 5 mg/mL was added and incubated for 4 h. Dimethyl sulfoxide (DMSO) in a volume of 100 µL was added into each well to dissolve the purple formazan formed by mitochondrial succinate dehydrogenase in viable cells. The colorimetric assay was measured and recorded at an absorbance of 570 nm using a plate reader (ELX 800, BioTek® Instruments, Inc. Winooski, VT, USA). Thus, the intensity of the colored product was directly proportional to the number of viable cells present in the culture. The percentage cell viability was calculated as $The\;percentage\;cell\;viability=\frac{A570\;of\;treated\;samples}{A570\;of\;the\;untreated\;sample}\times 100$

### 2.10. Statistical Analysis

Data obtained were expressed as mean ± standard error (M ± SE). The Shapiro–Wilk and Bartlett tests for homogeneity of variances were also used to ensure that response variables were normal. The mortality percentage of the larvae was analyzed using a two-way analysis of variance (ANOVA). Furthermore, the data on the inhibitory effect of *Xenorhabdus* sp. and *Photorhabdus* sp. bacteria on the viability of human lung fibroblast (WI-38) cells were analyzed using a one-way ANOVA. All analyses were conducted using the Minitab program [29]. Then, the *p*-value was adjusted according to Bonferroni correction to control the family-wise error rate, where *p* ≤ 0.05 means significance.

## 3. Results

### 3.1. Susceptibility of the Third-Instar Larvae of P. rapae to EPNs, H. bacteriphora and S. riobravis

**Figure 1:**
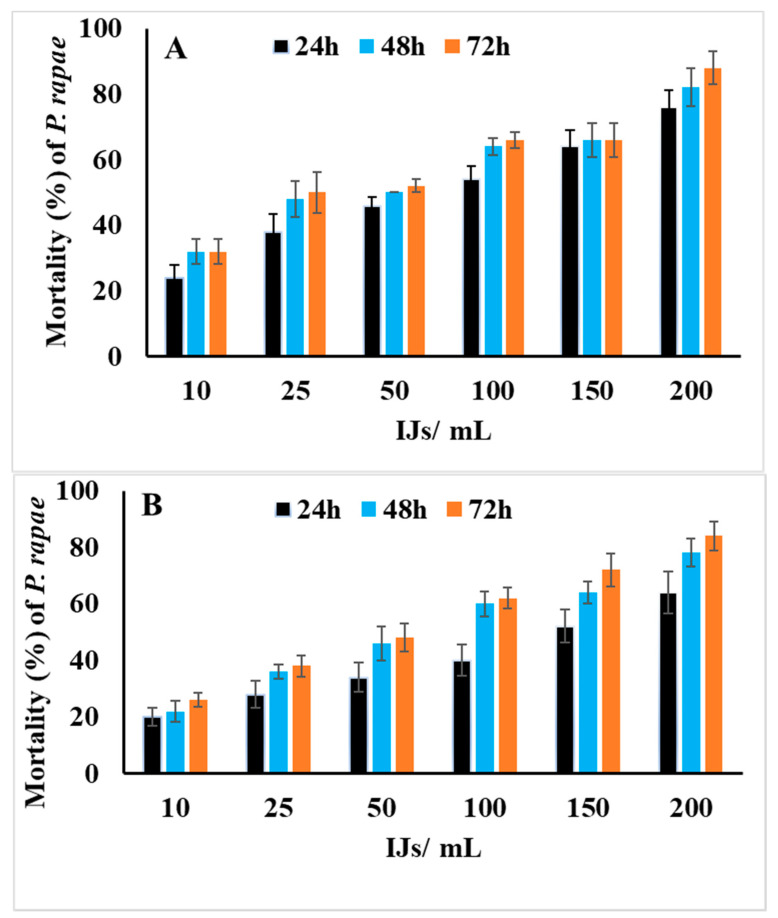
Mortality percentage (mean ± SE) of third-instar larvae of *P. rapae* exposed to different concentrations of *H. bacteriophora* (**A**) and *S. riobravis* (**B**) infective juveniles at different exposure periods. IJs/mL = infective juveniles/mL distilled water.

The data in Figure 1A, B show that both *H*. *bacteriophora* and *S*. *riobravis* had a highly significant effect on the mortality of *P*. *rapae* larvae (*p* < 0.001). The results showed that both nematode species induced a close percentage of mortality in *P*. *rapae* larvae (*p* < 0.05). Hence, *H*. *bacteriophora* induced 88% mortality, and *S*. *riobravis* induced 84% mortality at 200 IJs/mL distilled water and 72 h post exposure. The results also showed that a direct relationship existed between the percentage mortality and IJs’ concentration (*p* < 0.001). Thus, as the IJs’ concentration increased, the percentage of mortality increased. By contrast, exposure time did not significantly affect the percentage of mortality (*p* > 0.05).

### 3.2. Susceptibility of the Third-Instar Larvae of P. algerinus to EPNs, H. bacteriophora and S. riobravis

**Figure 2:**
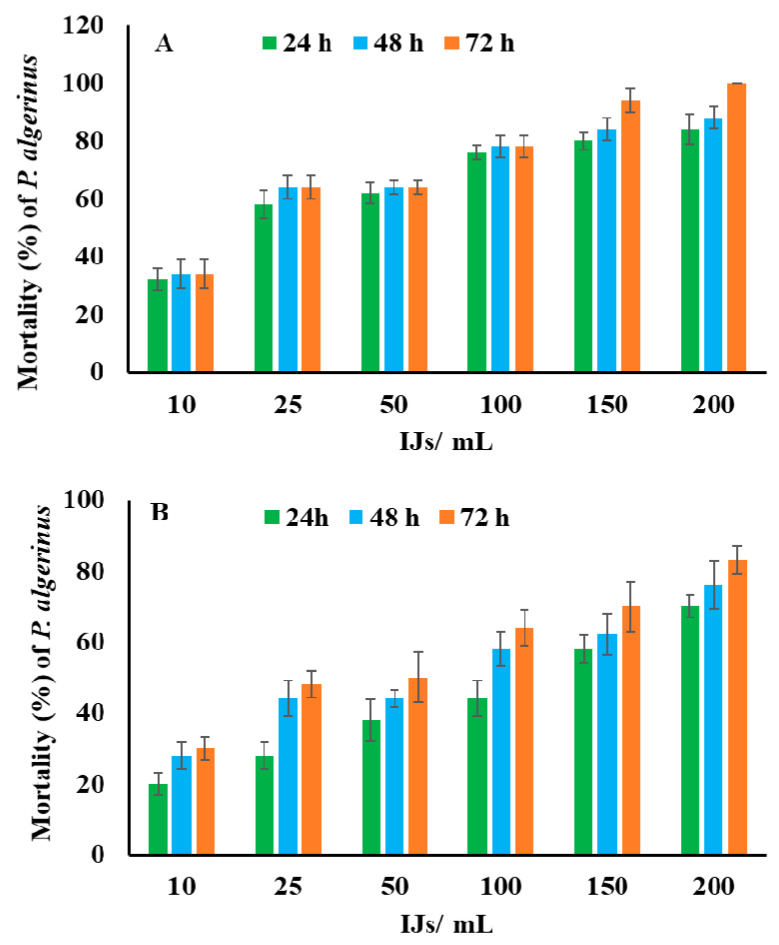
Mortality percentage (mean ± SE) of third-instar larvae of *P. algerinus* exposed to different concentrations of *H. bacteriophora* (**A**) and *S. riobravis* (**B**) infective juveniles at different exposure periods. IJs/mL = infective juveniles/mL distilled water.

The data in Figure 2A,B show that the third-instar larvae of *P*. *algerinus* were highly susceptible (*p* < 0.001) to both *H*. *bacteriophora* and *S*. *riobravis*. From the results, *H*. *bacteriophora* surpassed *S*. *riobravis* in inducing mortality in *P*. *algerinus*. As observed, *H*. *bacteriophora* induced 100% larval mortality compared with 83% induced by *S*. *riobravis* at 200 infective juveniles/mL distilled water and 72 h post exposure. The data also indicated that the mortality percentage had a direct relationship with the exposure time and IJs’ concentration (*p* < 0.001).

### 3.3. Lethal Concentration Values of EPNs, H. bacteriophora and S. riobravis, on the Third-Instar Larvae of P. rapae

The data in Table 1 show the LC_50_ and LC_90_ values of *H*. *bacteriophora* and *S*. *riobravis* against the third-instar larvae of *P*. *rapae*. The data show that at 24 and 48 h, *H*. *bacteriophora* was more effective against *P*. *rapae* larvae than *S*. *riobravis*, as it recorded lower LC_50_ and LC_90_ values of 56.88 and 1178.41 IJs/mL distilled water, respectively, at 24 h and; 35.52 and 948.28 IJs/mL distilled water, respectively at 48 h compared with *S*. *riobravis*, which recorded 125.39 and 4325.11 IJs/mL distilled water, respectively at 24 h, and; 50.15 and 1580.56 IJs/mL distilled water, respectively, at 48 h. However, at 72 h, no significant difference was observed between the LC_50_ and LC_90_ values of both nematode species. *H*. *bacteriophora* recorded 32.19 and 647.84 IJ/mL, respectively, compared with 35.14 and 606.22, respectively, for *S. riobravis*.

### 3.4. Lethal Concentration Values of EPNs, H. bacteriophora and S. riobravis, on the Third-Instar Larvae of P. algerinus

The data in Table 2 show that *H. bacteriophora* was more efficient against *P. algerinus* than *S. riobravis,* as it recorded a lower LC_50_ and LC_90_ of 22.79 and 365.36 IJs/mL distilled water at 24 h, 19.15 and 264.28 IJs/mL distilled water at 48 h, and at 72 h, it recorded 19.00 and 162.53 IJs/mL, respectively. *S. riobravis*, however, recorded 91.50 and 1927.89 IJs/mL distilled water at 24 h, 55.02 and 829.61 IJs/mL distilled water at 48 h, and at 72 h it recorded 43.50 and 547.12 IJs/mL distilled water, respectively. *P. algerinus* was more vulnerable to both nematode species than *P. rapae* according to the toxicity data in Table 1 and Table 2. In addition, the LC_50_ and LC_90_ values decreased with an increase in the time.

### 3.5. Morphological Characterization of the Isolated Symbiotic Bacteria, Photorhabdus sp. and Xenorhabdus sp.

**Figure 3:**
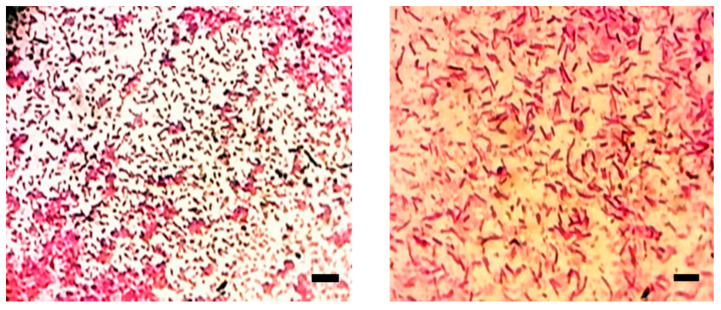
Photomicrograph of *Xenorhabdus* sp. cells (**left graph**) and *Photorhabdus* sp. (**right graph**) cells stained with Gram stain. Bar = 10 µm.

**Figure 4:**
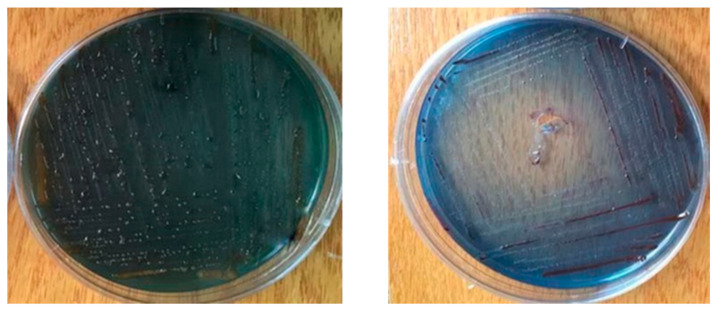
Photomicrograph of *Xenorhabdus* sp. cells (**left graph**) and *Photorhabdus* sp. (**right graph**) cells on NBTA medium.

**Figure 5:**
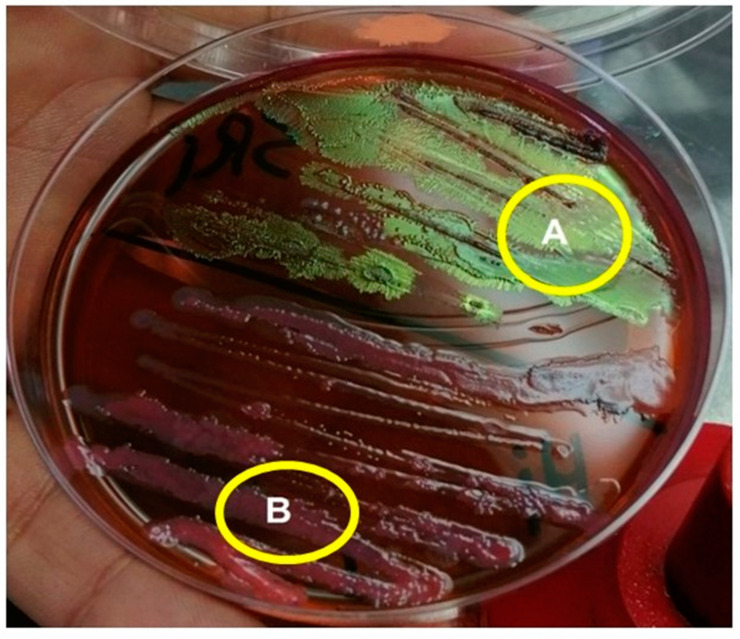
Photomicrograph of *Xenorhabdus* sp. cells (**A**) and *Photorhabdus* sp. cells (**B**) on EMB medium.

Based on the staining of the bacterial cells with Gram stain, it was obvious that both *Xenorhabdus* and *Photorhabdus* (Figure 3) bacterial cells had purple coloration. Meanwhile, the *Xenorhabdus* cells (left graph) were smaller than the *Photorhabdus* ones (right graph). Furthermore, on the basis of the growth of the tested bacteria on NBTA medium, the colony morphology of the *Xenorhabdus* bacterium was characterized as a dark blue, convex, and umbonate colony (Figure 4; left graph). However, *Photorhabdus* bacterium appeared as a dark red, convex, and umbonate colony (Figure 4; right graph). Additionally, using the EMB medium, *Xenorhabdus* bacterium was shown as flat, with a green metallic sheen colony (Figure 5A). By contrast, the *Photorhabdus* bacterium was demonstrated as a rose convex colony (Figure 5B).

### 3.6. Efficacy of the Symbiotic Bacteria, Xenorhabdus sp. and Photorhabdus sp., against Pieris rapae Larvae

**Figure 6:**
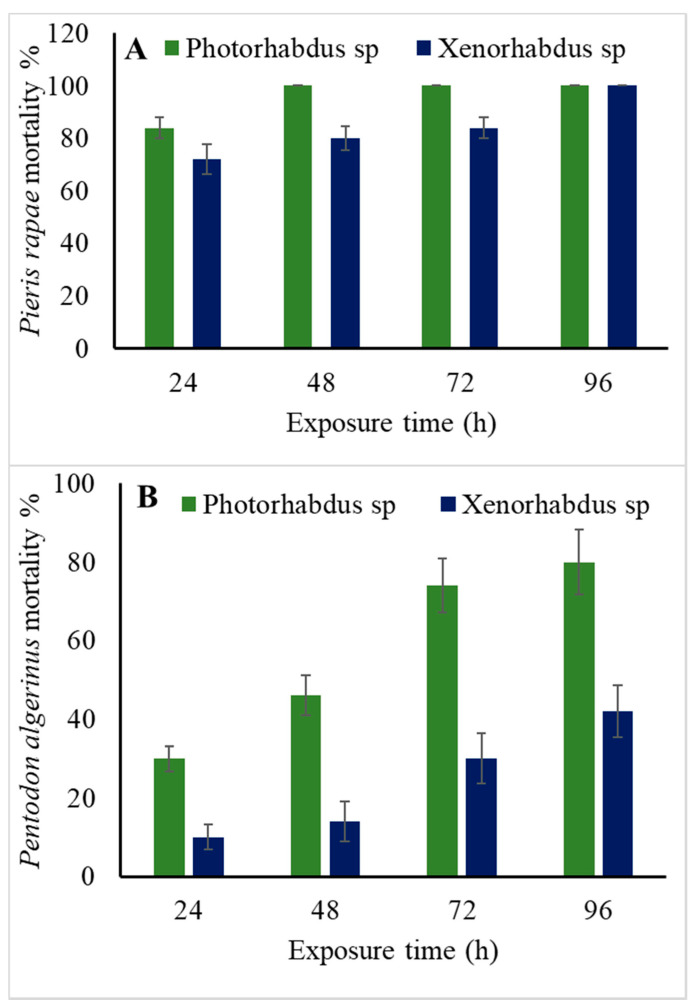
Mortality percentage (mean ± SE) of third-instar larvae of *P. rapae* (**A**) and *P. algerinus* (**B**) exposed to 3 × 10^7^ CFU/mL of symbiotic bacteria *Photorhabdus sp.* and *Xenorhabdus* sp. at different exposure times.

The data in Figure 6A show that both *Photorhabdus* sp. and *Xenorhabdus* sp. bacteria significantly affected *P*. *rapae* larvae (*p* < 0.001). Both bacterial species induced 100% larval mortality at 96 h of treatment. Moreover, the obtained data indicated that time had a significant effect (*p* < 0.01) on the percentage of mortality.

### 3.7. Efficacy of the Symbiotic Bacteria, Xenorhabdus sp. and Photorhabdus sp., against P. algerinus Larvae 

As shown in Figure 6B, both *Photorhabdus* sp. and *Xenorhabdus* sp. bacteria successfully induced mortality in *P*. *algerinus* larvae (*p* ˂ 0.001). *Photorhabdus* sp. bacterium caused 80% mortality and *Xenorhabdus* sp. induced 42% mortality at 96 h post exposure.

### 3.8. Efficacy and Time-Course Viability of Entomopathogenic Bacteria, Xenorhabdus sp. and Photorhabdus sp. against the Third-Instar Larvae of P. rapae under Field Conditions 

**Figure 7:**
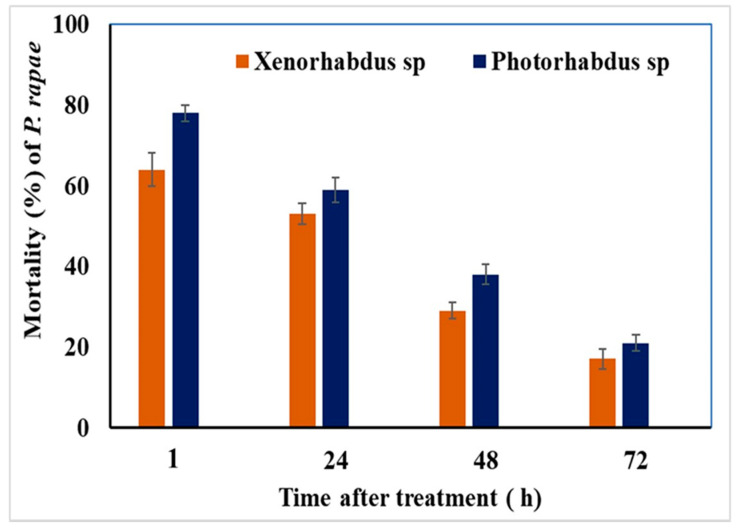
Mortality response (mean ± SE) of third-instar larvae of *P. rapae* fed on cabbage leaves treated with *Xenorhabdus* and/or *Photorhabdus* bacterial suspension at concentration of 3 × 10^7^ CFU/mL at different time intervals post application.

The data in Figure 7 show that both *Photorhabdus* sp. and *Xenorhabdus* sp. significantly controlled the third-instar larvae of *P*. *rapae* under field conditions (*p* < 0.001). However, the time-course viability of both bacteria was decreased by time. The highest mortality percentages were recorded in the sets where the larvae were exposed to cabbage leaves that were collected after one hour of application, and the lowest ones were recorded in the sets where the larvae were exposed to cabbage leaves that were collected 72 h post application, and this was for both bacterial genera. The data also indicated that *Photorhabdus* sp. bacterium was more effective than *Xenorhabdus* sp. bacterium, as it had the ability to induce 78, 59, 38, and 21% mortalities of the third-instar larvae of *P*. *rapae* at 1, 24, 48, and 72 h of application, respectively, compared with 64, 53, 29, and 17% at 1, 24, 48, and 72 h of application, respectively, for *Xenorhabdus* sp. bacterium. Furthermore, the mortality rates of the positive control (the leaf discs treated with media alone) were 1.00, 1, 0, and 0% at 1, 24, 48, and 72 h of application, respectively. The negative control (untreated leaves) mortalities were 0, 1, 0, and 0% at 1, 24, 48, and 72 h of the beginning of the experiment.

### 3.9. Gas Chromatography–Mass Spectrophotometry of Xenorhabdus sp. and Photorhabdus sp. Bacteria 

#### 3.9.1. *Xenorhabdus* sp. Bacterium

GC-MS analysis of *Xenorhabdus* sp. bacterium revealed 14 components (Table 3). The main constituent was 2-pyrrolidinone (35.04%), followed by 9-octadecenoic acid (z)-(oleic acid) (13.86%), 1,4-benzenediol, 2-(1,1-dimethylethyl)-5-(2-propenyl) (4.92%), 2,2-dideutero octadecanal (4.53%), octadecanoic acid (3.42%), 4-octadecenal (3.19%), cyclopentane tridecanoic acid, methyl ester (2.87%), 1,2-benzenedicarboxylic acid (2.80%), hexadecanoic acid (2.72%), 2,3-dihydroxypropyl ester paromomycin (2.63%), 1-tetradecanol (2.62%), 2,8,9-Trioxa-5-aza-1-silabicyclo [3.3.3]undecane, 1-methyl (2.37%), 7-nonenoic-7,8-d2 acid, methyl ester (2.11%), and docosanoic acid-1,2,3-propanetriyl ester (2.00%).

#### 3.9.2. *Photorhabdus* sp. Bacterium

The GC-MS of *Photorhabdus* sp. bacterium showed 12 components (Table 4). The main constituent was 2-piperidinone (44.09%), followed by pentadecanoic acid, 14-methyl-methyl ester (14.43%), 1,2-benzenedicarboxylic acid (13.20%), 1-eicosanol (5.57%), 4-trifluoroacetoxytetradecane (4.66%), bacteriochlorophyll-c-stearyl (2.91%), 15-methyltricyclo[6.5.2(13,14).0(7,15)] pentadeca-1,3,5,7,9,11,13-heptene (4.25%), octadecanoic acid, methyl ester (3.92%), bacteriochlorophyll-c-stearyl (2.91%), 1-tetradecanol (2.66%), 2(1h)-naphthalenone, octahydro-1-methyl-1-(2-p ropenyl)-, (1à,4aá,8aà) (2.28%), erucic acid (2.26%), and acetic acid, octyl ester (1.42%).

### 3.10. Morpho-Pathological Alterations in P. rapae and P. algerinus Larvae Caused by the Symbiotic Bacteria, Xenorhabdus sp. and Photorhabdus sp.

**Figure 8:**
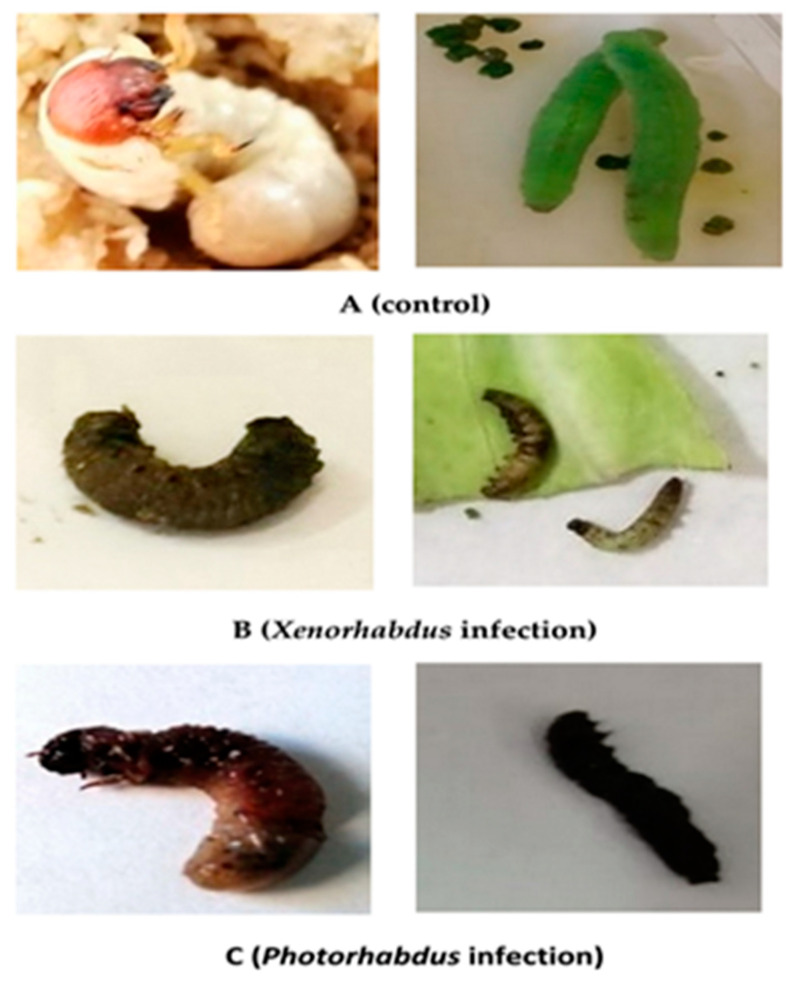
Photomicrograph of *P. algerinus* and *P. rapae larvae* control (**A**), infected with *Xenorhabdus* sp. bacterium (**B**), and infected with *Photorhabdus* sp. bacterium (**C**).

Figure 8 shows the morpho-pathological alterations of *P*. *rapae* and *P*. *algerinus* caused by *Xenorhabdus sp.* and *Photorhabdus* sp. bacteria. The control of *P*. *algerinus* larvae showed a creamy white coloration with a large brown head. However, *P*. *rapae* larvae had a bright green coloration (Figure 8A). Upon infection with *Xenorhabdus* sp. bacterium, the color of both insect species turned grayish (Figure 8B). Meanwhile, the color of *P*. *algerinus* and *P*. *rapae* larvae turned into a somewhat reddish color due to infection with the *Photorhabdus* bacterium (Figure 8C).

### 3.11. Cytotoxicity of the Isolated Symbiotic Bacteria, Xenorhabdus sp. and Photorhabdus sp.

In vitro, an MTT assay was conducted to evaluate the inhibitory effect of *Xenorhabdus* sp. and *Photorhabdus* sp. bacteria on normal WI-38 human cell viability. The results revealed a percentage cell viability of 85.3% for *Xenorhabdus* and 81.7% for *Photorhabdus* compared with 88.0% for the control (Table 5). Thus, these results reveal weak in vitro cytotoxicity of the tested bacteria on WI-38 cells (*p* ˃ 0.05).

## 4. Discussion

Various governments give special attention to the agricultural economy, because it is one of the most important sources of national income. Therefore, there is a great interest in agricultural pests and the damage they cause. Combating these pests has also become one of the most important priorities of people. For example, previous studies have been concerned with controlling *P*. *rapae;* however, they did not solve the problem. In addition, most of these studies focused on the use of chemical pesticides. Alternatively, studies on the biocontrol of *P*. *algerinus* remain scarce. Therefore, the present study aimed to evaluate the efficacy of *H*. *bacteriophora* and *S*. *riobravis*, including their symbiotic bacteria *Photorhabdus* sp. and *Xenorhabdus* sp., respectively, against *P*. *rapae* and *P*. *algerinus* larvae. The results revealed that both *H*. *bacteriophora* and *S*. *riobravis* nematodes successfully induced mortality in *P*. *rapae* and *P*. *algerinus* larvae. These results were in accordance with those of Ali et al. [30], who reported the efficacy of *Steinernema* masoodi, *Steinernema seemae*, *Steinernema carpocapsae*, *Steinernema glaseri*, and *Steinernema thermophilum* against *Helicoverpa armigera*, *G*. *mellonella*, and *Corcyra cephalonica*. Additionally, Reda et al. [16] reported that *S*. *carpocapsae* induced mortality in fourth-instar larvae and the pupae of *P*. *rapae*, with LC_50_ values of 18.148 and 38.96 IJs/larva and pupa, respectively. Recently, Askary and Ahmad [31] also recorded the efficacy of *Heterorhabditis pakistanensis* for controlling *Pieris brassicae*. Likewise, Grewal et al. [32] and Kleim et al. [33] improved the susceptibility of Japanese beetle, *Popillia japonica,* to EPNs infecting turf in the USA. WU [34] also reported the efficacy of *H*. *bacteriophora* and *H*. *megidis* against masked chafer white grubs, *Cyclocephala* spp. Similarly, Kajuga et al. [35] reported that both *H*. *bacteriophora* and *S*. *carpocapsae* killed up to 58% of white grubs. Another study also reported that *Steinernema abbasi* and *Heterorhabditis indica* had the capability to control the white grub *Leucopholis lepidophora* [36]. The obtained data also revealed that *H*. *bacteriophora* was more effective than *S*. *riobravis* against both *P*. *rapae* and *P*. *algerinus*. Shapiro-Ilan et al. [37,38] attributed the discrepancy in the infectivity and virulence of different EPN strains to different foraging behavior, host specificity, morphological characterization of the ENs, and the tolerance to host immune defenses.

Based on foraging behavior, EPNs have been classified into cruisers (active searchers) and ambushers (sit-and-wait foragers) [39]. Previous studies classified *Heterorhabditids* as cruisers and *Steinernematids* as ambushers [39]. Hence, the superiority of *H*. *bacteriophora* over *S*. *riobravis* in this study may be attributed to its foraging behavior as a cruiser. Grewal et al. [40] attributed the higher effect of *H*. *bacteriophora* and *H*. *megidis* than that of *S. carpocapsae* and *Steinernema scapterisci* to their different foraging behaviors. Additionally, Dillon et al. [41,42] reported that *S*. *carpocapsae* was less effective than the classic cruiser–foraging species, *Heterorhabditis downesi*.

EPNs’ morphological characterization is an important factor in determining their virulence toward insect hosts. The greater virulence of *H. bacteriophora* larvae compared to *S. riobravis* larvae may be attributable to the Heterorhabditid IJs’ distinctive buccal cuticular teeth, which facilitate their penetration into the host. Bedding et al. [43], who attributed the quick invasion rate of Heterorhabditid nematodes in several insect hosts to the dorsal tooth of their IJs, backed up this theory. This assumption could explain why Gouge et al. [44] and Menti et al. [45] discovered that Heterorhabditid nematodes infect insect hosts more quickly than Steinernematid nematodes. Furthermore, because Heterorhabditid nematodes are hermaphrodites, only one IJ is required to complete the life cycle and settle inside the insect host. *Steinernematids*, on the other hand, are amphimictic. As a result, for effective reproduction and establishment, both a male and a female would need to enter the host.

The variations in virulence between *H. bacteriophora* and *S. riobravis* against *P. rapae* and *P. algerinus* could potentially be attributed to their tolerance of host immune responses. This finding agrees with that of Silva et al. [46], who reported that in *Manduca sexta*, *P*. *luminescens* cells accompanied with *H*. *bacteriophora* secreted antiphagocytic and anti-encapsulation factors that permitted nematodes to overcome the insect’s immune defenses.

The obtained data also revealed that the third-instar larvae of *P*. *algerinus* were more susceptible to *H*. *bacteriophora* and *S*. *riobravis* infestation than those of *P*. *rapae*. The appreciable differences in the susceptibility of the two insect hosts may be attributed to different host morphological structures, sizes, behaviors, and immune defense mechanisms. This opinion is in agreement with that of Medeiros et al. [47]; they attributed the differences in the susceptibility of different stages of *Pseudaletia unipuncta* to *S*. *carpocapsae*, *S*. *glaseri*, and *H*. *bacteriophora* to different insect sizes, behaviors, and immune defense mechanisms. As a result, *P. algerinus’* larger size may be the explanation for its superiority as a nematode host over *P. rapae*. Similarly, Lewis et al. [48] found that large larvae are more attractive to EPNs than smaller larvae. Boff et al. [49] also found that as larval size rose, the amount of invading *H. megidis* IJs and the production of IJs from infected wax moth and vine weevil larvae increased. Another reason for the *P. algerinus* larvae’s higher vulnerability to nematodes than that of the *P. rapae* larvae is that the *P. algerinus* larvae reside deep in the soil, where nematodes live. As a result, infection is thought to have occurred once or more, and the nematodes recognized the insect’s immunity map. The *P. rapae* larvae, however, reside on the surface of the cabbage plant, so it is probable that the infestation occurred for the first time; thus, the immune system’s tools worked together to combat the nematode onslaught.

As shown in the above result, *H*. *bacteriophora* and *S*. *riobravis* to some extent succeed in the control of both *P*. *algerinus* and *P*. *rapae*. However, it is known that symbiotic bacteria have a major role in killing insects. Hence, we isolated the symbiotic bacteria of *H*. *bacteriophora* and *S*. *riobravis* and then applied them to control both insect species. Subsequently, the symbiotic *Xenorhabdus* sp. and *Photorhabdus* sp. from *S*. *riobravis* and *H*. *bacteriophora*, respectively, were isolated, mass cultured, and applied at a concentration of 3 × 10^7^ CFU/mL against *P*. *algerinus* and *P*. *rapae*. The obtained data revealed that both *Xenorhabdus* sp. and *Photorhabdus* sp. significantly affected *P*. *algerinus* and *P. rapae* larvae. Some studies have also emphasized the ability of *Xenorhabdus* spp. and *Photorhabdus* spp. to induce mortality in different insect species [8,18,50,51,52,53].

The data obtained also revealed that *Photorhabdus* sp. was more effective than *Xenorhabdus* sp. against both *P*. *algerinus* and *P*. *rapae*; however, *P*. *rapae* was more susceptible. This higher lethality of *Photorhabdus sp.* than that of *Xenorhabdus* sp. correlates with the better efficacy of *H*. *bacteriophora* than that of *S. riobravis*. These results were in line with those of Rahoo et al. [51], who reported that the mortality caused by *P*. *luminescens* was significantly higher than that of *X*. *bovienii*. Moreover, ref. [8] reported that *Photorhabdus* species produced 75–96% mortality in *S*. *frugiperda* larvae. In contrast, *Xenorhabdus* bacteria were less active, with mortality rates in the range of 33–57%.

The insecticidal activity of *Photorhabdus* sp. and *Xenorhabdus* sp. bacteria may be attributed to the fact that both produce toxin complexes, proteases, lipases, lipopolysaccharides, and other active components [46,54,55,56,57]. These components make caterpillars floppy [58], induce apoptosis, inhibit hemocyte motility, and inhibit cellular and humoral immunity [59,60].

The GC-MS analysis of *Xenorhabdus* sp. and *Photorhabdus* sp. bacteria revealed that *Xenorhabdus* sp. bacterium possessed 14 main components, whereas *Photorhabdus* sp. bacterium had 12 main components as shown in Table 3 and Table 4, respectively. Five of these compounds (2-Piperidinone, 1,2-benzenedicarboxylic acid, tetradecanol, and octadecanoic acid) were commonly detected in the two bacterial genera. However, the ratios in *Photorhabdus* sp. were higher than those in *Xenorhabdus* sp.

The piperidinone compound was the highest ever in both *Photorhabdus* sp. and *Xenorhabdus* sp. bacteria. Piperidinone is an organic chemical that is a derivative of piperidine. Piperidine, on the other hand, is a colorless fuming liquid with an ammoniacal, peppery odor. Piperidine is a common chemical reagent and building block in the production of organic molecules, including pharmaceuticals. The piperidine structural motif is present in numerous natural alkaloids. [59,60]. Vivekanandhan et al. [61] emphasized the role of piperidinone in the insecticidal activity of *Beauveria bassiana* against *Cx*. *quinquefasciatus* mosquito.

Several other studies have detected similar compounds from different strains of *Xenorhabdus* and *Photorhabdus* bacteria [62,63,64,65]. These compounds may be responsible for the insecticidal activity of *Xenorhabdus* and *Photorhabdus* bacteria in this study. This assumption may be supported by the opinion of Ullah et al. [62], who attributed the insecticidal and antimicrobial activity of *P*. *temperate* against *G*. *mellonella* larvae to 1,2-benzenedicarboxylic acid, which plays a crucial role in the inhibition of insect phenoloxidase (the key mediator of insect immune systems). Similarly, Hemalatha et al. [66] attributed the insecticidal activity of *X*. *nematophilus* against *Ferrisia virgata* to 1,2-Benzenedicarboxylic acid and cosine groups. Hasan et al. [64] also attributed the virulence of six *X*. *nematophila* strains against *Spodoptera exigua* to active secondary compounds, such as benzeneacetic acid, n-Decanoic acid, Tetradecane,1-Decene, and 3-Benzylidene-hexahydro-pyrrolo, which inhibit the insect immune system. Later, Mollah and Kim [65] detected fatty alcohol, 1-ecosine, heptadecane, octadecanes, and methyl-12-tetradecen-1-ol acetate in different strains of *Xenorhabdus* and *Photorhabdus* bacteria. The authors suggested that these compounds inhibited the insect’s phospholipase A2, thereby eradicating the insect immune system. The phospholipase A2 enzyme catalyzes fatty acids that are later oxygenated by cyclooxygenase and lipoxygenase enzymes to produce prostaglandins and leukotrienes, respectively, which are mediators of the immune response in insects [67]. This was supported by the findings of [68], who reported that *X*. *nematophila* and *P*. *temperata* were responsible for suppressing the phospholipase A2 enzyme. Another compound identified from the GC-MS analysis of *Photorhabdus* sp. in this study was uric acid, which plays a crucial role as a food inhibitor in order to prevent infected insects from feeding, thus inducing insect death.

In the continuation of this study and in an attempt to model an integrated idea regarding the efficacy of the tested EPNs and their symbiotic bacteria, we evaluated the efficacy of *Xenorhabdus sp.* and *Photorhabdus* sp. bacteria to control *P*. *rapae* in the field. The data obtained showed that both bacterial species significantly decreased the population of *P*. *rapae* in the field. The percentage mortality reached 78% by *Photorhabdus* sp. and 64% by *Xenorhabdus* sp. Although there are several studies documenting the use of EPNs for insect control in the field [31,69,70,71,72,73,74,75,76], those that document the efficacy of *Xenorhabdus* sp. and *Photorhabdus* sp. bacteria in the field are scarce. Gerritsen et al. [77] recorded the efficacy of *Photorhabdus* and *Xenorhabdus* strains against *Frankliniella occidentalis* and *Thrips tabaci* after sucking the bacteria from treated leaves. Therefore, these results from the efficacy of *Xenorhabdus* sp. and *Photorhabdus* sp. in the field confirm the results at the laboratory scale and are further proof of the effectiveness of these bacteria.

## 5. Conclusions

From this study, we concluded that *H*. *bacteriophora, S*. *riobravis*, and their symbiotic bacteria (*Photorhabdus* sp. and *Xenorhabdus* sp., respectively) are effective candidates for biocontrolling *P*. *rapae* and *P*. *algerinus*, either in experimental or field studies. The results also clarified that both symbiotic bacteria can be utilized separately from their nematodes. Thus, we can recommend these EPNs and their symbiotic bacteria to be certified alternatives for chemical pesticides in the control programs of *P*. *rapae* and *P*. *algerinus* and to be tested against other insect pests.

## Figures and Tables

**Table 1:** Response of third-instar larvae of *P. rapae* to EPNs, *H. bacteriophora,* and *S. riobravis*.

EPNs	Exposure Period (h)	LC_50 (95%FL)_	LC_90 (95%FL)_	Slope
*Heterorhabditis bacteriophora*	24	56.88 (26.26–123.25)	1178.41 (543.86–2553.30)	0.90
	48	35.52 (15.43–81.75)	948.28 (412.03–2182.44)	0.97
	72	32.19 (15.11–68.57)	647.84 (304.18–1379.76)	1.01
*Steinernema riobravis*	24	125.39 (50.63–310.56)	4325.11 (1746.38–10711.65)	0.83
	48	50.15 (20.96–119.98)	1580.56 (660.61–3781.61)	0.86
	72	35.14 (16.95–72.83)	606.22 (292.51–1256.36)	1.05

LC_50_ and LC_90_: lethal concentration kills 50 and 90%, respectively, of insect host. Concentration expressed as infective juveniles/mL distilled water.

**Table 2:** Response of third-instar larvae of *P. algerinus* to EPNs, *H. bacteriophora*, and *S. riobravis*.

EPNs	Exposure Period (h)	LC_50_	LC_90_	Slope
*Heterorhabditis bacteriophora*	24	22.79 (10.89–47.68)	365.36 (174.67–764.23)	1.06
	48	19.15 (9.37–39.12)	264.28 (129.37–539.90)	1.12
	72	19.00 (9.82–35.24)	162.53 (90.09–293.22)	1.43
*Steinernema riobravis*	24	91.50 (41.93–199.68)	1927.89 (883.43–4207.20)	0.974
	48	55.02 (27.37–110.61)	829.61 (412.69–1667.74)	1.09
	72	43.50 (22.59–83.77)	547.12 (284.10–1053.61)	1.17

LC_50_ and LC_90_: lethal concentration kills 50 and 90%, respectively, of insect host. Concentration expressed as infective juveniles/mL distilled water.

**Table 3:** Gas chromatography–mass spectrophotometry analysis of *Xenorhabdus* sp. bacterium.

Peak No.	Rentation Time	Area%	Compound Name	Molecular Formula
1	5.63	2.11	7-NONENOIC-7,8-D2 ACID, METHYL ESTER	C_10_H_16_D_2_O_2_
2	5.84	2.63	Paromomycin	C_23_H_45_N_5_O_14_
3	7.48	35.04	2-PYRROLIDINONE	C_4_H_7_NO
4	8.87	4.53	2,2-DIDEUTERO OCTADECANAL	C_18_H_34_D_2_O
5	12.81	2.62	1-TETRADECANOL	C_14_H_30_O
6	13.19	2.37	2,8,9-Trioxa-5-aza-1-silabicyclo[3 .3. 3]undecane, 1-methyl-	C_7_H_15_NO_3_Si
7	15.66	4.92	1,4-benzenediol, 2-(1,1-dimethylethyl)-5-(2-propenyl)-	C_13_H_18_O_2_
8	16.89	3.19	4-Octadecenal	C_18_H_34_O
9	22.92	2.87	CYCLOPENTANETRIDECANOIC ACID, METHYL ESTER	C_19_H_36_O_2_
10	23.97	13.86	9-OCTADECENOIC ACID (Z)-(Oleic Acid)	C_18_H_34_O_2_
11	24.06	2.72	hexadecanoic acid, 2,3-dihydroxypropyl ester	C_19_H_38_O_4_
12	27.06	3.42	OCTADECANOIC ACID	C_18_H_36_O_2_
13	31.98	2.80	1,2-benzenedicarboxylic acid	C_24_H_38_O_4_
14	35.28	2.00	Docosanoic acid, 1,2,3-propanetriyl ester	C_69_H_134_O_6_

**Table 4:** Gas chromatography–mass spectrophotometry analysis of *Photorhabdus* sp. bacterium.

Peak Number	Rentation Time	Area%	Compound Name	Molecular Formula
1	6.39	1.42	ACETIC ACID, OCTYL ESTER	C_10_H_20_O_2_
2	7.51	44.09	2-Piperidinone	C_5_H_9_NO
3	8.30	13.20	1,2-benzenedicarboxylic acid	C_8_H_6_O_4_
4	12.81	2.66	1-TETRADECANOL	C_14_H_30_O
5	15.63	4.25	15-METHYLTRICYCLO[6.5.2(13,14).0(7,15)]PENTADECA-1,3,5,7,9,11,13-HEPTENE	C_16_H_14_
6	16.31	2.28	2(1H)-NAPHTHALENONE, OCTAHYDRO-1-METHYL-1-(2-P ROPENYL)-, (1à,4Aá,8Aà)-	C_14_H_22_O
7	16.89	4.66	4-Trifluoroacetoxytetradecane	C_16_H_29_F_3_O_2_
8	20.30	5.57	1-EICOSANOL	C_20_H_42_O
9	22.22	2.91	Bacteriochlorophyll-c-stearyl	C_52_H_72_MgN_4_O_4_
10	22.93	14.43	PENTADECANOIC ACID,14-METHYL-, METHYL ESTER	C_17_H_34_O_2_
11	26.10	3.92	OCTADECANOIC ACID, METHYL ESTER	C_19_H_38_O_2_
12	27.06	2.26	Erucic acid	C_22_H_42_O_2_

**Table 5:** Percentage viability of WI-38 human cells treated with the isolated *Xenorhabdus* sp. and *Photorhabdus* sp. bacteria.

Treatments	Percentage Viability of WI-38 Human Cells (%)
*Xenorhabdus* sp.	85.33 ± 1.52
*Photorhabdus* sp.	81.66 ± 3.05
Control (samples treated only with medium)	88.00 ± 4.00

## Data Availability

All data are presented within the article.
